# Supplementary material for: Discovery and pharmacophoric characterization of chemokine network inhibitors using phage-display, saturation mutagenesis and computational modelling
Source: Nat Commun. 2023 Sep 16;14:5763. doi: 10.1038/s41467-023-41488-z (PMC10505172; doi:10.1038/s41467-023-41488-z)
Supplement: Supplementary file 4 — Source Data [file 41467_2023_41488_MOESM4_ESM.zip › SourceData/scripts/xoctet_20230808081637.tar!/xoctet/analysis/kinetic/SuppTable4.docx]

Table 4: Biolayer Interferometry Analysis. Expt ID indicates independent
experiments. KD[M]: Mean KD (molar). SE: standard error. N: number of dose
responses with good fits used to calculate summary statistics.

| Expt ID | Sample | Analyte | KD [M] | KD:SE [M] | N |
| --- | --- | --- | --- | --- | --- |
| E12 | sHD2 | CCL8 | 1.425e-07 | 4.94e-08 | 5 |
| E32 | sHD2 | CCL8 | 8.49e-08 | 1.90e-08 | 4 |
